# Beneficial Effects of Spiritual Experiences and Existential Aspects of Life Satisfaction of Breast and Lung Cancer Patients in Poland: A Pilot Study

**DOI:** 10.1007/s10943-022-01601-w

**Published:** 2022-06-24

**Authors:** Marcin Wnuk

**Affiliations:** Department of Work and Organizational Psychology, Adam Mickiewicz University, Szamarzewskiego Street 89AB, 60-568 Poznań, Poland

**Keywords:** Breast cancer, Lung cancer, Spiritual experiences, Hope, Meaning in life, Life satisfaction, Mediator, Moderator, Structural equation modeling

## Abstract

Spiritual and existential issues are important factors for oncology patients' well-being. This study aimed to examine the beneficial role of spiritual experience, hope, and meaning in life for life satisfaction in patients diagnosed with breast and lung cancer. It was hypothesized that spiritual experiences and life satisfaction are indirectly related through hope, as well as meaning in life mediates the relationship between hope and life satisfaction. It was a pilot study with a sample consisting of 4 men and 46 women, 24–83 years of age oncology patients. The following measures were used: Cantril Ladder, Purpose in Life Test, Herth Hope Index as well as Daily Spiritual Experiences Scale. A sample of Polish patients with breast and lung cancer confirmed the beneficial effects of spiritual experiences and existential aspects of life for their life satisfaction. According to obtained results, hope was indirectly related to life satisfaction through meaning in life. Also, spiritual experiences were positively indirectly related to life satisfaction through the pathway of hope and meaning in life. Theoretical and practical implications of the achieved results were discussed.

## Introduction

On the one hand, Poland has a low rate of cancer incidence (Ferlay et al., 2010). On the other hand, Poland’s 1- and 5-year survival rate was the lowest among the countries in the Eurocare study (Coleman et al., 2008). For lung and breast cancer patients, Poland’s mortality rate is one of the largest among European countries (Organisation for Economic Cooperation and Development [OECD] 2010). In Poland, there is a growing interest to find and recognize resources responsible for cancer patient's survivorship and their better well-being. Recent research has emphasized the significant role of religious-spiritual and existential issues in oncology patients' quality of life (Gayatri et al., 2021; Ownsworth & Nash, 2015). In Poland, the impact of religiousness on the mental health of cancer patients was explored (Krok et al., 2019; Zarzycka et al., 2019) indicating indirect mechanisms underlying this relationship. There is a lack of research regarding the benefits of spiritual experiences and existential needs for oncology clients regardless of their religious commitment. This study aimed to fill this gap by exploring the role of spiritual experiences and existential needs such as meaning in life and hope for the life satisfaction of Polish oncology patients and verification of the mechanisms underlying this process. Spiritual experiences are the core of spirituality and religiousness, resulting from religious commitment (Wnuk, 2021a, 2021b), and/or secular practices inspired by secular worldviews, such as meditation and contemplation (Galen, 2018; Labelle et al., 2015). Spirituality and religiousness are different but similar and overlapping phenomena (Baumsteiger & Chenneville, 2015; Hyman & Handal, 2006), but spirituality is recognized as a broader construct encompassing religious commitment as a one form of spiritual expression (Baumsteiger & Chenneville, 2015; Hyman & Handal, 2006) called religious spirituality in contrast to secular spirituality (Wnuk, 2021a).

Also, Daily Spiritual Experiences Scale (DESE) used in this research emphasizes that spiritual experiences can have their roots in religion in the case of religiously inclined individuals and spirituality (beyond religion) about non-believers. It consists of items regarding spiritual qualities that came from both religion and spiritual involvement – “I find my strength in my religion or spirituality,” “I find comfort in my religion or spirituality” (Underwood, 2011). This measure has a universal character and is insensitive to the religious denomination, which means that it can be applied to different research groups with different religious backgrounds, as well as non-believers. In the text, the term religious-spiritual refers to both religious and spiritual aspects of functioning. Regarding religious individuals encompass both religious and spiritual facets of existence, but for non-believers, only spiritual aspects related to secular values. Also, spiritual experiences were considered without recognizing their religious or spiritual sources of origin. It didn't matter, especially since every research participant was religious, and a probability for both of these possibilities existed.

Spiritual experiences can have a beneficial influence on well-being based on spiritual mechanism underlying this relationship through existential needs, such as meaning in life and hope or positive emotions (Galen, 2018). Spiritual experiences can fill many functions in adjustment to illness through fostering hope and facilitating in finding meaning in experiencing pain, physical and psychological limitations, and needs frustration (Gall & Grant, 2005). The theoretical framework for the study was Frankl’s (2009) meaning in life conception and Seligman's (2002) theory of happiness. Frankl (1975), in his approach, emphasized the beneficial role of spirituality and religion in finding meaningful and purposeful life and treated them as sources of hope. Seligman (2002), inspired by Aristotle's internal spirit called “Eudajmon” which personified human talents, values, and dispositions, underlines that life in coherence with his suggestions leads to a fruitful and meaningful life as a way to achieve happiness. The relationship between hope and meaningful life can be considered within the conception of “broaden and build of positive emotions” (Fredrickson, 2001). Hope as one of the positive emotions, defined as a looking forward to the future and expecting the best to happen, results in a broadening of experience and the building of a wide repertoire of adaptation resources and facilitates finding the meaning (Fredrickson & Branigan, 2005).

In this study, these functions were verified in a sample of oncology patients based on assumptions that their spiritual experiences are positively correlated with hope, which in turn directly and indirectly through finding meaning is related to life satisfaction.

### Spirituality/Religiousness and Well-Being – Hope as a Mediator

Religious-spiritual facets of life can be supportive with struggle with cancer patients' daily stress, leading to stress-related and posttraumatic growth (Casellas-Grau et al., 2017; Park et al., 2009) better well-being (Holt et al., 2011; Krok et al., 2019). Gayatri et al. (2021) in their review of literature have confirmed positive relationships between different measures of religiousness and spirituality and different aspects of the quality of life of cancer patients. Also, the positive role of hope, not only in cancer, for subjective well-being is well-documented (Pleeging et al., 2019). Empirical analysis conducted by Pleeging et al. (2019) has indicated connections between hope and life satisfaction, happiness, positive as well as negative affect, which was independent of the tool used to measure hope. For example, in the longitudinal study in a sample of oncology patients from the USA, hope was associated with a lower level of depression, less somatic symptoms, and higher intensity of positive affect (Rabkin et al., 2009).

Some researchers have suggested that in oncology patients religious-spiritual aspects of life are indirectly related to mental health via enhancing hope, effective religious/spiritual coping, receiving social support, and finding purpose and meaning in life (Crane, 2009). Recently these potential religious-spiritual mechanisms in this group of patients have been tested regarding religious/spiritual coping (Holt et al., 2011; Krok et al., 2019), social support (Lim & Yi, 2009), and hope (Zarzycka et al., 2019). For example, in a Zarzycka et al. study (2019) in a sample of individuals suffering from cancer religious comfort positively correlated with hope, which in turn predicted lower anxiety. Scioli et al. (2016) in their conception of fundamental hope have linked spiritual needs with hope of cancer patients. According to this approach, hope is derived from four human needs: spirituality, mastery, survival, and attachment. The spiritual need of hope encompasses inter alia empowered or strengthened by God or higher power/force, felt close to, or connected with God or higher power/force or discovered or reminded of goodness in the world or the universe. This research has indicated that spiritual resources of hope besides social resources are important, especially for those dealing with late-stage breast cancer.

The mediating role of hope in the relationship between religiousness/spirituality and wellbeing was confirmed in studies among adolescent (Chang et al., 2016; Nell & Rothmann, 2018; Wnuk & Marcinkowski, 2014; Wnuk, 2021a). For example, in a sample of Polish and Chilian students more frequent spiritual experiences were positively associated with hope, which in turn predicted higher life satisfaction and higher intensity of positive affect (Wnuk, 2021a; Wnuk & Marcinkowski, 2014).

The second purpose of this study was the verification of the spiritual mechanism underlying the relationship between spiritual experiences and life satisfaction and the role of hope in this relation.

#### Hypothesis 1

In a sample of Polish oncology patients, spiritual experiences are indirectly related to life satisfaction through hope.

### Role of Existential Needs for Cancer Patients’ Well-Being

The role of existential needs as an important issue in oncology patients is widely recognized (Henoch & Danielson, 2009; Ownsworth & Nash, 2015; Rabkin et al., 2009). Purpose in life and hope are components of existential well-being that includes realizing values, having goals, controlling one’s destiny, and finding self-acceptance (Baumeister et al., 2002). Questions about one’s purpose in life and hope have become meaningful especially in difficult or crisis situations like a cancer diagnosis. When people have to cope with a cancer diagnosis, the situation forces them to search for the meaning or purpose of the diagnosis in their life (Breitbart, 2002). They must struggle with the fear of death and other negative emotions such as despair, anger, or sadness (Bowes et al., 2002) and find the strength to defeat a disease.

In one study, six major themes repeatedly emerged as essential components of psycho-spiritual well-being oncology patients: self-awareness, coping and adjusting effectively to stress, relationships and connectedness with others, sense of faith, sense of empowerment and confidence, and living with meaning and hope (Lin & Bauer-Wu, 2003).

Among 258 cancer patients located in urban areas, 51% declared a desire to receive help in overcoming fear, 42% wished to find hope, and 40% were concerned about finding and purpose in life (Moadel, et al., 1999). Women with ovarian tumors found that purpose in life and hope were important issues influencing their feelings of well-being or sense of despair (Bowes et al., 2002).

The crucial role of meaning in life in oncology patients' recovery proves that many therapeutic interventions focus on this phenomenon in improving the quality of life of this population (Breitbart et al., 2010, 2012; Henry et al., 2010; Mok et al., 2012). In the longitudinal study, Scheffold et al. (2015) have proven that the therapy focus on searching for meaning and hope, positively influences health, reducing depression, fatigue, distress and improving quality of life and spiritual well-being. Based on meta-analysis conducted of 15 studies involving 14 controlled trials, Oh and Kim (2014) have discovered that spiritual interventions have a positive impact on meaning in life, anxiety, spiritual well-being, and depression.

According to recent research, purpose and meaning in life, as well as hope, were positively associated with cancer patients' quality of life (Bluvol & Ford-Gilboe, 2004; Kang et al., 2017; Tomich & Helgeson, 2002; Weebers, 2006). For example, Kang et al. (2017) have confirmed that in a sample of nonmetastatic breast cancer survivors both feeling hopeful and have a purpose in life were positively connected with happiness.

Among patients suffering from prostate cancer, hope was positively related to positive affect and happiness, as well as negatively correlated with depression and negative affect (Blank & Bellizzi, 2005). In a study of 999 newly diagnosed patients with different types of cancer meaning in life positively correlated with physical, social, psychological and functional well-being (Whitford & Olver, 2012).

Meaning in life and hope are closely connected constructs (Feldman & Snyder, 2005). Regardless of the plethora of conceptions of meaning in life and hope in literature has emphasized that they are multidimensional, future-orientated and goal-directed (Feldman & Snyder, 2005; Herth, 1991; Miller & Powers, 1988; Scioli et al., 2016; Yalçın & Malkoç, 2015). Following the Feldman and Snyder's (2005) suggestions in Frankl theory, goals can be treated as values, to which people endeavor to find meaning and purpose in life. According to Frankl (2009), there are three types of values—creative, experiential, and attitudinal. Creative values are actualized by creating or producing something like writing a paper, erecting a building, etc. Experiential values are actualized using senses when someone sees, touches, tastes, smells, hears, or in another way experiences something. Attitudinal values are actualized when someone is in a very difficult life situation like a fatal disease and no longer is able to create or experience but regardless of that still can have meaning and purpose in his life suffering from dignity. In this kind of situation are oncology patients because they suffer a lot trying to find meaning and purpose in disease and have a chance to suffer with a dignity. Also in Emmons's theory (2003), goals are essential elements for a meaningful life, which are attained through achievement/work, relationship/intimacy, religion/spirituality, and self-transcendence/generativity. Goals are a central component of hope but in contrast to optimism hope as a multidimensional dynamic life force assumes not an only expectation in achieving the good, which is personally significant, but also engagement in attaining this purpose.

In the literature is a noticeable lack of research examining the role of existential needs in the mechanism underlying positive outcomes in cancer survivors. Most studies have focused on the positive role of hope or meaning in life on well-being without searching for the potential interactive effect of both of these variables. Also is observed the lack of research testing the meaning in life as an antecedent of hope in the relationship with the well-being or inversely meaning in life as a mediator in the relationship between hope and well-being (Feldman & Snyder, 2005). This deficit is especially noticeable in the area of oncology. Some suggestions on this topic have appeared in recent studies, but their results are inconclusive. Feldman and Snyder (2005) conducted a factor analysis and regression analysis by used different measures of meaning in life and hope as well as anxiety and depression as dependent variables to prove that hope is a central component of life meaning but achieving results were ambiguous. Exploratory factor analysis has supported the hypothesis that hope is a part of a larger factor, which is meaning in life, but the results regarding hope as a mediator in the relationship between meaning in life and anxiety were nonconclusive. In regression analysis, the strength of the relationship between hope and anxiety, controlled by meaning in life, was statistically reduced. Also, the predictive power of meaning in life for anxiety was statistically significantly reduced when hope was introduced to regression analysis. This situation took place for every meaning in life indicators. What is worth emphasizing noticed that meaning in life moderated the relationship between hope and depression. The relationship between hope and depression was stronger in group of participants with a lower level of meaning. In the literature have appeared some suggestions which indicated that hope is rather antecedent than the result of finding meaning in life (Allan, 2015; Ferguson et al., 2017; Nell, 2014; Yang & Wu, 2021). For example, hope predicted meaning in life in older adults (Ferguson et al., 2017) and Chinese nurses (Yang & Wu, 2021). On the other hand, in another study, both hope and meaning in life as correlated variables explained mindfulness (Nell, 2016). The strength of the relationship between meaning in life and mindfulness was stronger than the strength of the relationship between hope and mindfulness, which could mean that meaning in life mediated the relationship between hope and mindfulness.

#### Hypothesis 2

In a sample of Polish oncology patients, meaning in life mediated the relationship between hope and life satisfaction.

## Methods

### Sample and Procedure

The initial number of patients consisted of 100 patients from settings treated for cancer at the Warsaw Central Clinical hospital and the Warsaw Amazonki Club collaborating with the Cancer Centre in the Warsaw Institute of Cancer at Wawelska Street. Only one criterion of admission was used such as diagnosis of breast or lung cancer. Patients were examined with a standardized set of questions between January 11, 2008, and December 31, 2008. Among 100 patients, 15 were too sick to participate, 26 declined to participate, and 9 patients did not completely fill the questionnaires, resulting in the research remained 50 patients.

### Measures

The following research measures were applied: Daily Spiritual Experiences Scale (DSES), Purpose in Life Scale (PIL), Herth Hope Index (HHI) and Cantril Ladder.

Daily Spiritual Experiences Scale (DSES) is a tool to measure ordinary experiences of connection with the transcendent. It has satisfied validity and reliability. Participants answer the 16 questions on a continuum between 1 and 6 (1—many times a day, 2—every day, 3—most days, 4—some days, 5—once in a while, 6—never or almost never) (Underwood, 2011).

The Purpose in Life Test (PIL) consists of 20 statements regarding the need for meaning in life. Each question is answered on the continuum between 1 and 7, with 7 indicating maximum intensity related to the meaning in life, and 1 indicating a minimum intensity. The reliability of this tool measured by the coefficient of correlation (r-Pearson) was 0.82 with the Spearman–Brown correction = 0.90 (Crumbaugh & Maholic, 1964).

Hope Herth Index (HHI) is a tool that has good psychometrical properties (Nayeri et al., 2020). This measure is dedicated especially to clinical patients. Participants answered 12 questions expressed on the 4-step Likert-type scale (Herth, 1992).

Cantril Ladder verified life satisfaction. It is a simple and understandable measure for all groups of respondents. The Cantril Ladder consists of one question in which the research participants evaluate life satisfaction on a scale from 0 (minimum) to 10 (maximum), life satisfaction (Cantril, 1965). This tool is characterized by satisfying reliability (Czapiński 1992).

### Conceptual Model

To better understand the beneficial role of filing spiritual and existential needs for cancer patients' well-being, it was decided to verify spiritual and existential mechanisms underlying their life satisfaction. It was assumed that hope as a result of spiritual experiences was indirectly, through finding meaning in life, related to life satisfaction. According to Diener’s concept of subjective well-being containing cognitive and affective well-being dimensions (Diener & Ryan, 2009), only cognitive indicator of this construct regarding the evaluation of life satisfaction was used.

The two different models were examined through path analysis by using the maximum likelihood method of SEM. This statistical solution is dedicated to the variables which have normal distribution. The decision to use the maximum likelihood method was due to the fact that all research variables' values of skewness and kurtosis fitted in the interval between -2 to 2 (see table), which was the proof that data can be considered normally distributed (George, & Mallery, 2010). To test whether the models are fitted to the data arbitrary indicators were chosen treated in the literature as an appropriate measure of fitting, such as Root Mean Square Error of Approximation (RMSEA), Normed Fit Index (NFI), Goodness of Fit index (GFI), and Comparative Fit Index (CFI). Due to the small research sample, bootstrapping methods were used with 5000 bootstrap resamples and 95% interval confidence. Both bias-corrected (BC) percentile method for 95% confidence intervals was derived. When the values of upper level (UL) and lower level (LL) do not include 0, the test statistic is significantly different from zero (Preacher & Hayes, 2008).

Paths between variables were marked by arrows. Based on Bovero et al. study (2019) indicating the lack of correlation between spiritual experiences and the “meaning” dimension of spiritual well-being in oncology patients, it was assumed that spiritual experiences will not be directly related to purpose and meaning in life but only indirectly through hope.

Verification of additional model and introduction of other statistical methods were dictated of inconsistency in the literature about the role of meaning in life and hope for well-being and attempt to more precisely and more complex consider this issue. This activity was used to answer some questions. First, whether meaning in life mediates in the relationship between hope and life satisfaction. Secondly, whether hope and life satisfaction is indirectly positively related through meaning in life. Thirdly, whether meaning in life moderates the relationship between hope and life satisfaction.

## Results

**Scheme 1.**
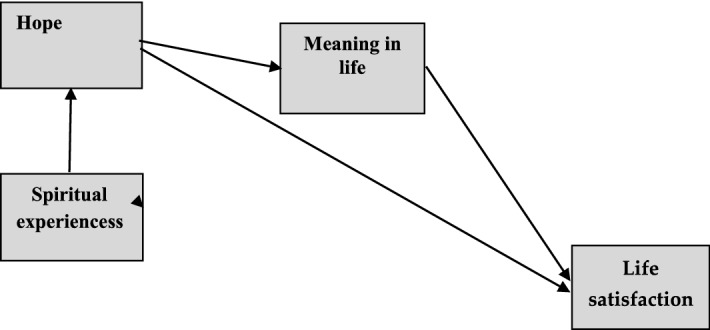
Model of relationships between variables consistent with the research hypotheses. *Source*: author’s research

Demographics variables are presented in Table 1, descriptive statistics in Table 2, and values of fit models indicators in Table 3. The model of relationships between variables consistent with the research hypotheses is shown in Scheme 1.

**Table 1:** Demographics variables (*n* = 50)

	Classification	Percentage or mean
Gender	Men	8.5%
	Women	91.5%
Age		55.16 years
Diagnosis	Lung cancer	8.5%
	Breast cancer	91.5%
Education	Primary education	16%
	Occupational education	8%
	High school education	44%
	University education	32%
Duration of disease		2.12 years

**Table 2:** Descriptive statistics (*n* = 50). *Source*: author’s research

	DSES	PIL	HHI	Cantril Ladder
Mean	63.52	106.36	37.50	5.91
Standard deviation	20.49	20.68	8.14	2.553
Skewness	− 0.64	− 1.02	− 0.63	− 0.45
Kurtosis	− 0.40	0.46	0.38	− 0.74
Minimum	19	51	23	0
Maximum	96	138	48	10
Reliability	0.97	0.93	0.91	–

Daily Spiritual Experience Scale; *PIL* Purpose in Life Test, *HHI* Herth Hope Index, Cantril Ladder—life satisfaction

**Table 3:** Fit indicators fot testing models (*n* = 50). *Source*: author’s research

Number of models	CMIN/DF	RMSEA	CFI	GFI
Model 1	.496 (*p* = 0.796)	[0.000, 90% (0,000; 0.233)]	1	0.97
Model 2	.555 (*p* = 0.519)	[0.000, 90% (0,000; 0.256)]	1	0.98

Model 1 Spiritual experiences are related to hope, which is indirectly related to life satisfaction through meaning in life

Model 2 Spiritual experiences are related to hope, which mediates the relationship between meaning in life and life satisfaction

To verify if meaning in life moderates the relationship between hope and life satisfaction Process macro in SPSS was applied (Hayes, 2018).

**Scheme 2.**
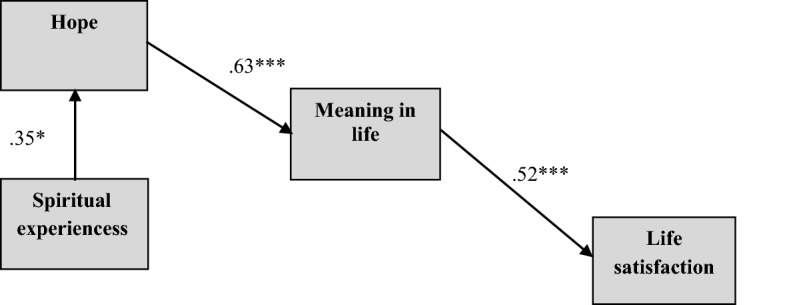
Final model best fitted to data. *Note*. The standardized regression coefficients are presented. *p < .05, **p < .01, ***p < .001. *Source*: author’s research

There were no statistically significant moderating effect of meaning in life in the relationship between hope and life satisfaction (CI 95% [LL =—0.0034; UL = 0.0035], moderating effect = 0.001, *t* = 0.049; *p* = 0.961). This meant that meaning in life is not a moderator in the relationship between hope and life satisfaction (Scheme 2).

In models 1 and 2, the RMSEA indicator value was below the acceptable level of 0.06, the CFI value was higher than the required 0.95 (Hu & Bentler, 1999), and GFI was higher than treated as a minimum 0.9 (Byrne, 1994) (see Table 3). This meant that they were well-fitted to the data (see Table 3), but there one exception. In model 2, direct path between hope and life satisfaction was not statistically significant (CI 95% [LL = -0.123; UL = 0.524], beta = 0.218, *p* = 0.187). In model 1 presented on Scheme 1 spiritual experiences predicted hope (CI 95% [LL = 0.065; UL = 0.589], beta = 0.351, *p* ≤ 0.05), and hope predicted meaning in life (CI 95% [LL = 0.391; UL = 0.778], beta = 0.634, *p* ≤ 0.01). Additionally, meaning in life was directly related to life satisfaction (CI 95% [LL = 0.277; UL = 0.707] beta = 0.528, *p* ≤ 0.01). In this model, spiritual experiences were indirectly related to life satisfaction through the pathway hope-meaning in life (CI 95% [LL = 0.027; UL = 0.256], indirect effect = 0.117 *p* ≤ 0.05), as well as hope was indirectly related to life satisfaction through meaning in life (CI 95% [LL = 0.146; UL = 0.516], indirect effect = 0.334, *p* ≤ 0.01). Additionally spiritual experiences were indirectly related to meaning in life through hope (CI 95% [LL = 0.043; UL = 0.496], indirect effect = 0.222, *p* ≤ 0.05).

## Discussion

The study's main purpose was to examine spiritual and existential mechanisms underlying life satisfaction in oncology patients suffering from breast and lung cancer. Additionally tested the relationship between hope and meaning in life in explaining life satisfaction among members of this group.

Contrary to Wnuk’s (2021c), Feldman and Snyder’s (2005) studies did not find the moderating effect of meaning in life in the relationship between hope and life satisfaction.

The hypothesis regarding the indirect relationship between spiritual experiences and life satisfaction through hope was only partially confirmed. According to previous studies conducted on Polish samples, spiritual experiences correlated positively with hope (Wnuk, 2021c; Wnuk & Marcinkowski, 2014), but inconsistently with assumptions hope was not directly related to life satisfaction, only indirectly through finding meaning. These differences can be explained by the fact that in one of these studies relationship between hope and meaning in life in explaining life satisfaction and affectivity was not explored (Wnuk & Marcinkowski, 2014). Only separate indirect pathways between spiritual experiences and well-being indicators through hope or meaning in life were tested. In the second study, the relationship between hope and feeling of hopelessness depended on the level of meaning in life and was not significant in a group of participants with a higher than average level of meaning in life and negative in the groups of participants with average and less than average level of meaning in life (Wnuk, 2021c).

According to previous studies, spiritual experiences were positively related to hope and the same as in Bovero et al. research (2019) were not directly related to finding meaning and purpose. It is worth noticing that inconsistent with obtained results in other samples research from Poland, such as students or Alcoholics Anonymous spiritual experiences, was moderately correlated with finding meaning in life (Wnuk & Marcinkowski, 2014; Wnuk, 2021c). This discrepancy can be explained by two reasons.

The first one refers to the indirect relationship between spiritual experiences and meaning in life via hope. It was confirmed that in oncology patients from Poland suffering from breast and lung cancer spiritual experiences are positively connected with a higher level of hope, which in turn facilitates finding meaning in life.

The second explanation regards using by oncology patients other values as a framework for finding meaning in life, such as social support, preserving human values and ideals, optimism, and feeling financially secure (Scheffold et al., 2014; Shao et al., 2014). Non-believers can satisfy their existential needs using secular sources through building a stable worldview based for example on evolution or natural selection (Galen, 2018). Also spiritual experiences are not only a domain of individuals with religious worldview (Galen, 2018). This study did not verify whether the spiritual growth of cancer patients was the result of religious or secular values. Statistics show that Poles are engaged in religious practices, and religion is a very significant value in their life (Pew Research Center, 2018). It is possible that for those oncology patients for whom religion is a central value in their life, religion is a framework, which in these difficult circumstances such as living with cancer leads to reconstructing the meaning of negative events (Pargament & Park, 1995), understanding and integrating those experiences (Acklin et al., 1983). This benevolent effect of religion is seen in religious nations, where the socialization of religious faith, the same as in Poland, is a common phenomenon (Lun & Bond, 2013). Recent research has emphasized the role of existential needs in this mechanism. For example, in a Chilian student spiritual experiences were indirectly related to life satisfaction (Wnuk, 2021a). The same, Diener et al. (2011), in their research on a sample of individuals from 154 nations have confirmed the mediating role of meaning and purpose in life in the relationship between religiousness and life satisfaction. Also, other secular values and worldviews can serve as a cognitive-behavioral function facilitating finding meaning in life. In a sample of Alcoholics Anonymous (AA), beyond religion, involvement in this form of support filled this role, being related to a meaningful and purposeful life (Wnuk, 2021b).

The hypothesis, which referred to meaning in life as a mediator in the relationship between hope and life satisfaction, was partially confirmed. According to obtained results in a sample diagnosed with breast and lung cancer, hope is associated with finding meaning in life, which in turn is related to better life satisfaction. It was proof that the indirect relationship between hope and life satisfaction through the meaning in life is more possible than the mediation of hope between meaning in life and life satisfaction. This meant that only one of these existential variables is a predictor of oncology patients’ life satisfaction. These results were not consistent with the research where hope and meaning in life separately predicted anxiety and depression (Feldman & Snyder, 2005) as well as mindfulness (Nell, 2016). On the other side, it was confirmed that consistent with the recent studies hope probably facilitates finding meaning in life (Allan, 2015; Ferguson et al., 2017; Nell, 2014; Yang & Wu, 2021).

It was confirmed that hope is a facilitator of finding purpose and meaning in life. Indirect relationship between hope and life satisfaction through meaning in life can be explained following Seligman’s ways leading to happiness and "broaden and build a theory of positive emotions" (Fredrickson, 2001). Besides hedonic and engagement ways to happiness, Seligman describes the eudaimonic way to life contentment as a result of a meaningful and purposeful life. In oncology, an idea that meaning and purpose in life mediate between some psychosocial variables and well-being was materialized in a study by Shao et al. (2014). Consistent with results of the current study, in a sample of elderly stroke survivors in Chinese communities relationships between social support as well as optimism and subjective well-being were indirect through hope (Shao et al., 2014). Also, Frankl’s “tragic optimism” (2009) can serve as an interpretative framework for achieved results. Tragic optimism takes a place when despite negative circumstances, such as living with cancer full of pain and suffering, can be transformed into something meaningful. Living with cancer is a possibility to actualize Frankl’s attitudinal values (2009), to reduce helplessness, maintain and foster hope, and finally find meaning and purpose as a significant factor for life satisfaction. In a similar vein, Fredrickson in her "broaden and build a theory of positive emotions" has emphasized that hope as a positive emotion is a positive antecedent of meaningful and purposeful life due to the development of cognitive resources which facilitates finding the meaning of the events and situations (Fredrickson, 2001).

In this research, the role of existential variables such as meaning in life and hope in the relationship between spiritual experiences was tested. According to obtained results, the spiritual experiences of cancer patients have indirectly related to life satisfaction through the pathway of hope and meaning in life. It means that more spiritual experiences are positively correlated with hope, which in turn facilitate finding meaning in life, which finally are related to higher life satisfaction.

According to results in Polish patients suffering from breast and lung cancer, pathway between spiritual experiences and life satisfaction leads through two existential variables such as hope and meaning in life.

The achieving results have theoretical and practical implications. Mechanisms underlying the life satisfaction of cancer patients based on spiritual and existential issues were positively verified. Obtained results have essential implications in nursing, medicine, and psychology encouraging representatives of these fields to support oncology patients in filling their existential and spiritual needs as important aspects of their life satisfaction. For example, according to Baumeister, successfully finding meaning in life fulfills four main needs: purpose, values, sense of efficacy, and self-worth (2002). Professionals working with cancer patients should concentrate on those needs. Previous research has confirmed that spiritual (Oh & Kim, 2014) and meaning-focused interventions (Breitbart et al., 2010, 2012; Henry et al., 2010; Mok et al., 2012) have a beneficial influence on cancer patients.

## Study Limitations

This conducted study has several limitations. The main limitation is the small study sample size. Using bootstrapping method was the solution, which to some extent balanced it. Considering that the research model included four variables, using SEM for path analysis was reasonable. There were more than 12 participants for one variable, and it was consistent with Nunnally's suggestion that there should be more than 10 research participants per variable (1967).

Due to the specifics of the sample, which is determined by the nature of breast cancer as the dominant disease among women, the generalizability of this study is limited mainly to Polish women who have breast cancer living in urban areas. The present study was cross-sectional, due to which the relationships between the variables used cannot be presented in the cause and effect order. On the other hand, the used method indicates the direction of the variables. It could be interesting to conduct a longitudinal study among this population using additional religious and secular variables, which could lead to spiritual growth and satisfy existential needs among oncology patients diagnosed with breast cancer.
